# Breastfeeding among South Sudanese refugees in protracted settlements in Adjumani district, Uganda: facilitators and barriers

**DOI:** 10.1186/s13006-023-00549-1

**Published:** 2023-03-17

**Authors:** Christine N. Walters, Hasina Rakotomanana, Joel J. Komakech, Margaret Kabahenda, Jillian M. Joyce, Deana A. Hildebrand, Lucia Ciciolla, Barbara J. Stoecker

**Affiliations:** 1 Department of Nutritional Sciences, Oklahoma State University, Stillwater, OK USA; 2 Department of Food Science, Nutrition, and Health Promotion, Mississippi State University, Starkville, Mississippi USA; 3 Department of Food Technology and Nutrition, Makerere University, Kampala, Uganda; 4 Department of Psychology, Oklahoma State University, Stillwater, OK USA

**Keywords:** Breastfeeding, Facilitators, Barriers, South Sudanese refugees, Uganda

## Abstract

**Background:**

Evidence suggests that forced migration and refugee status may adversely impact mothers’ breastfeeding choices. Furthermore, suboptimal breastfeeding practices have been reported among vulnerable populations including those living in refugee settlements. Therefore, this study investigated the barriers and facilitators of breastfeeding in protracted settlements in Adjumani district, in the West Nile region in Uganda.

**Methods:**

This study was conducted among refugees living in protracted settlements located in Uganda in July 2019. Participants, originally from South Sudan, included mothers (*n* = 63) and fathers (*n* = 32) of children less than 24 months of age. Agojo, Ayilo-I, and Nyumanzi were randomly selected among the 17 refugee settlements in Adjumani. Participants formed a total of six focus group discussions (FGDs); four FGDs for mothers and two FGDs for fathers. Each FGD consisted of 15–16 participants. Data were transcribed verbatim and back-translated into English. Thematic analysis was used and data were analyzed using NVivo, v. 12.

**Results:**

Facilitators of breastfeeding included knowledge of breastfeeding benefits, support from husband/father, support from the community, and support from non-governmental organizations. Mothers and fathers noted that breastfeeding protected children from diseases and breastfed children grew well. Fathers, the community, and organizations provided material support for breastfeeding mothers. Four themes were identified as barriers to breastfeeding: physical, socioeconomic, knowledge, and psychosocial. Mothers and fathers described physical barriers such as mothers stop breastfeeding when they are sick or they feel they are not producing enough breastmilk. Mothers reported that working or educated mothers may use other milk to feed their infant. Some mothers and fathers believed infants under six months needed more than breastmilk. Fathers described psychosocial barriers such as mothers’ fear of pain during breastfeeding and maternal mental health issues.

**Conclusion:**

Interventions and policies that aim to improve breastfeeding in protracted settlements should consider addressing the barriers to breastfeeding at each level: physical, socioeconomic, knowledge, and psychosocial. Involving and encouraging support from husbands/fathers, relatives, and the community may increase adherence to breastfeeding recommendations.

## Background

Optimal nutrition is important for healthy infant growth and development [1]. Breastmilk is considered a key component of adequate nutrition because it contributes to decreased risk of infectious diseases and infant mortality, while also promoting optimal neurodevelopment [1, 2]. Additionally, breastfeeding has long-term benefits including reduction in the risk of chronic diseases such as cardiovascular disease, diabetes and obesity [3]. The risks of undernutrition, infectious disease, and mortality are significantly increased among refugee children less than 24 months of age who are not breastfed and in infants less than six months of age who are not exclusively breastfed [4]. The World Health Organization (WHO) recommends that mothers breastfeed within the first hour of birth, exclusively breastfeed until the infant is six months of age and continue breastfeeding to two years or beyond [5]. The Global Breastfeeding Scorecard has breastfeeding targets to be reached by 2030 including early initiation of breastfeeding (70%), exclusive breastfeeding (70%), and continued breastfeeding (80%) [6]. Despite the WHO breastfeeding recommendations, global breastfeeding rates remain low, especially in vulnerable populations, such as those who have been displaced or are refugees living in another country. Globally, over 80 million individuals have been forcibly displaced, with 26.3 million of those individuals being refugees in another country [7]. The South Sudanese refugee population is the third largest globally and is the largest refugee crisis in Africa [8]. In 2011, after a distressing civil war, South Sudan declared independence and became the youngest country in the world [9]. However, since 2013 when armed conflict broke out, over 2.3 million South Sudanese have fled their home country to find refuge [8, 9]. Over 80% of South Sudanese refugees are women and children [8]. Most of these refugees have fled to neighboring countries of Sudan, Ethiopia, Kenya, Democratic Republic of Congo, and Uganda [8]. Uganda hosts more South Sudanese refugees than any other country and nearly 65% of all the refugees in Uganda have fled South Sudan [9]. Most refugees in Uganda live in settlements and more than half of the refugees reside in northern Uganda or West Nile [9]. Adjumani district is located in the West Nile region of Uganda and hosts over 200,000 refugees in settlements, among whom 60% are children [10]. In 2020, the three leading morbidities among children under 5 were malaria (41%), respiratory tract infections (23%), and acute watery diarrhea (12.7%) [10].

Limited research has been conducted on breastfeeding in protracted settlements in Uganda; however, a recent study in Adjumani district reported that among 561 mothers in these settlements, just over half (57%) breastfed their infants within one hour of birth [11]. In 2020, early initiation of breastfeeding rates ranged from 58 to 84.4% across refugee settlements. Exclusive breastfeeding rate across all settlements was 62.3%, a considerable decrease from the previous exclusive breastfeeding rate of 90.7% reported in 2014. Across settlements in Adjumani, the exclusive breastfeeding rate was 42.3%, nearly 20% points lower than the average across all settlements. Compared to other districts in the West Nile region, breastfeeding rates of refugees were the lowest in Adjumani [10].

Recent studies are consistent with the WHO’s concern that forced migration and refugee status may adversely impact mothers’ breastfeeding choices [12]. In Rwanda, a study among refugees revealed only 34.4% of infants were exclusively breastfed for the first six months of life, despite 74.4% of mothers demonstrating knowledge of and having positive attitudes towards exclusive breastfeeding [13]. A meta-ethnographic study of refugee and migrant women’s experiences of breastfeeding concluded that mothers who did not have access to traditional postpartum practices and who experienced tension with breastfeeding in a new country were more likely to cease breastfeeding [14]. Therefore, the barriers and facilitators of breastfeeding were investigated among refugees living in protracted settlements in the West Nile region of Uganda. The two main research questions for this qualitative study were: (1) what are the facilitators of breastfeeding among women living in protracted settlements? and (2) what are the barriers to breastfeeding among women living in these settlements? The data will be useful for informing policies and interventions aimed at improving breastfeeding among refugees.

## Methods

### Participants

This study was conducted in protracted settlements located in Uganda in July 2019. Participants, who were originally from South Sudan and living as refugees in the settlements, included mothers (*n* = 63) and fathers (*n* = 32) of children less than 24 months of age. Agojo, Ayilo-I, and Nyumanzi were randomly selected among the main refugee settlements in Adjumani. Along with Lutheran World Federation community service officers and health care workers in the randomly selected settlements, the research team coordinated with the Village Health Teams, who routinely interact with parents, to prepare lists of mothers and fathers of children under 2 years of age living within the villages where they operate. From those lists, participants were selected randomly, and with the help of the VHTs, prospective participants were mobilized for the FGDs. Mothers and fathers were recruited from different households. There were a total of six focus group discussions (FGDs): four FGDs for mothers and two FGDs for fathers and each FGD had 15–16 participants.

### Data collection

Questions used in the FGDs (Table 1) were developed based on previous research and were reviewed by the research team and doctoral committee. Before data collection, the questions were examined by research translators who were hired for their proficiency in English as well as in at least one of the three local languages. The translated documents were cross-checked by two other translators and pre-tested among parents in the settlements. The final translated FGD guide was agreed upon by the research team. FGDs were conducted by the research team along with enumerators, who were hired based on their prior experience in bridging communication gaps between humanitarian organizations and refugees. FGDs were held in community centers and were conducted in Arabic, Dinka, or Madi, the languages primarily spoken by the participants and were audio-recorded. Probing questions were used during the FGDs and saturation was considered to be achieved when no new information was contributed by participants.

**Table 1:** Discussion guides for mothers’ and fathers’ focus group discussions

**Mothers’ focus group discussion guide**
What do you think are the benefits of breastfeeding? At what age should children stop breastfeeding? What might a parent give a child to eat before they are 6 months old? How does your community support breastfeeding mothers? What are some reasons why mothers do not breastfeed their children? What are some reasons children are given food before six months old?
**Fathers’ focus group discussion guide **
What do you think are the benefits of breastfeeding? At what age should children stop being breastfed? What might a parent give a child to eat before they are six months old? How does your community support breastfeeding mothers? What are some reasons why mothers do not breastfeed their children? What are some reasons children are given food before they are six months old? What do you feel is your role in supporting your wife who is breastfeeding?

### Data analysis

Audio recordings of the FGDs were transcribed verbatim by native speakers of Arabic, Dinka, and Madi. Next transcriptions were back-translated to English by native speakers who were also proficient in English. Data were coded by the research team using NVivo, v. 12 [15]. Cleaning of codes was completed by the primary investigator and reviewed by the research team. The research team identified codes with similarities to form themes that appeared to answer the research questions using thematic analysis. When discrepancies in analysis occurred among the researchers, they were resolved as a team during coding and thematic analysis. However, due to the nature of the participants’ responses, there was minimal disagreement among researchers during the data analysis.

### Trustworthiness/reliability

Data were collected from participants living in three different protracted settlements which allowed for multiple perspectives and which likely increased data reliability. Probing questions used during the FGDs helped achieve saturation. Furthermore, during the formation of codes and themes, the involvement of the research team further reinforced trustworthiness and reliability.

### Ethics

Approval was obtained from the Makerere University School of Health Sciences Research and Ethics Committee in Uganda (SHSREC REF: 2019-020), the Uganda National Council of Science and Technology (SS 5038) and the Institutional Review Board at Oklahoma State University (HS-19-2). Local permission was acquired from the Ugandan Office of the Prime Minister (OPM/R/107). Eligible participants were made aware of the purpose of the study and were provided the opportunity to ask questions. Participation in the study was voluntary and all participants signed informed consent before data collection began. Compensation for participation was provided to all participants in the form of household and food items that were worth approximately 1.5 USD.

## Results

### Demographic characteristics of participants

Key descriptive characteristics of the participants are summarized in Table 2. The mean age of fathers (39.7 years) was higher than that of mothers (27.1 years). Fewer mothers reported working outside the home (14.3%) compared to fathers (50%). A higher percentage of mothers were illiterate (36.5%) than fathers (18.8%). Many mothers (71.5%) reported attending four or more antenatal sessions.

**Table 2:** Descriptive characteristics of study participants (n = 95)

**Characteristics**	**Mothers (n = 63)**	**Fathers (n= 32)**
	**Mean**	**Mean**
Respondent age (years)	27.1	39.7
Child’s age (months)	13.2	16.5
Household size	7.3	8.8
Number of living children	3.5	5.7
	**n (%)**	**n (%)**
Occupation		
No occupation	53 (86)	16 (50)
Farmer	0	4 (13)
Office work	4 (6)	0
Other	6 (8)	12 (37)
Ethnicity		
Dinka	31 (49)	27 (84)
Madi	27 (43)	3 (9)
Other	5 (8)	2 (7)
Education level		
Illiterate	23 (37)	6 (19)
Informal education	1 (1)	2 (6)
Formal education	39 (62)	24 (75)
Duration as refugee in West Nile, Uganda		
2 - 3 years	48 (76)	12 (39)
4 - 5 years	11 (18)	11 (37)
> 5 years	4 (6)	9 (24)
Antenatal visit attendance		
Less than four	18 (29)	-
Four or more	45 (71)	-

### Facilitators

Beliefs and knowledge about breastfeeding benefits, support from husband/father, support from the community, and support from non-governmental organizations (NGOs) were identified facilitators of breastfeeding (Table 3). Many mothers and fathers mentioned that breastmilk was nutritious and would protect against disease as well as help the child grow strong and well.

*“An infant fed with breastmilk grows well and will be strongly protected from the diseases.” - Mother (FGD Nyumanzi)*

*“Breastmilk makes the baby strong and sometimes protect the baby from diseases.” - Father (FGD Ayilo-I)*

*“An infant fed with breastmilk will grow well.” – Mother (FGD Nyumanzi)*

*“Breastmilk makes the baby strong.” – Father (FGD Ayilo-I)*

**Table 3:** Facilitators of breastfeeding

Themes	Codes	Quotations	Data source (# of times mentioned)
**Beliefs and knowledge about breastfeeding benefits**	Child will be bright	“Breastmilk develops a clever mind or brain of the baby.” - Mother (FGD Agojo) “The child will be bright.” - Mother (FGD Agojo)	Mothers FGD (2)
	Child will grow well and strong	“Breastmilk makes the baby fat. So when other people come visit, they feel happy when they see the baby.” - Mother (FGD Nyumanzi) “When a baby is fed on breastmilk, the baby becomes very strong.” - Mother (FGD Nyumanzi) “Breastmilk makes the baby feel strong.” – Mother (FGD Nyumanzi) “Breastmilk makes a child grow stronger.” - Mother (FGD Agojo) “The child will be very strong.” - Mother (FGD Agojo) “The child will be strong.” - Mother (FGD Agojo) “A child breastfeeding makes them strong.” - Father (FGD Ayilo-I) “Breastmilk builds the baby’s body.” - Father (FGD Ayilo-I) “Breastmilk makes the baby grow strong and healthy.” - Father (FGD Ayilo-I)	Fathers FGD (2) Mothers FGD (7)
	Breastmilk is nutritious and protects from disease	“Breastmilk protects the baby.” - Mother (FGD Nyumanzi) “Breastmilk protects the baby from diseases.” - Mother (FGD Nyumanzi) “Breastmilk makes the baby strong and sometimes protects the baby from diseases.” - Father (FGD Ayilo-I)	Fathers FGD (1) Mothers FGD (3)
**Support from husband/father**	Provides food	"The husband should come up with the money to buy food such as meat so that the lactating mother gets soup.” - Mother (FGD Nyumanzi) “You try to maintain your wife through feeding so that she can’t be weak.” - Father (FGD Ayilo-I) “You ensure that the mother has eaten.” - Father (FGD Ayilo-I) “We help out wife in some ways like providing food.” - Father (FGD Ayilo-I)	Fathers FGD (4) Mothers FGD (1)
	Provides emotional support	“The husband makes sure she’s happy.” - Father (FGD Ayilo-I) “The love we have for them that is how we help them with breastfeeding.” - Father (FGD Ayilo-I)	Fathers FGD (3)
	Do household chores	“When it is rainy season the man has to cultivate in the garden at home.” - Father (FGD Ayilo-I)	Fathers FGD (2)
**Support from community**	Grandmother provides food	“In our homes, grandmothers provide breastfeeding mothers with food for cooking so that she can produce more breastmilk for the child.” - Mother (FGD Agojo)	Mothers FGD (1)
	Neighbors provide materials	“And also some neighbors support the breastfeeding mother with providing them firewood and drinking water.” - Mother (FGD Agojo) “Also when the man is poor to provide for the baby and the breastfeeding mother it is his role to borrow from the neighbors.” - Father (FGD Ayilo-I)	Fathers FGD (1) Mothers FGD (1)
**Support from non-governmental organizations (NGO)**	NGO provides education	“And also Plan International was educating mothers about breastfeeding.” - Mother (FGD Agojo)	Mothers FGD (1)
	NGO provides food	“They were being supported by the partners giving out flour for porridge MTI and Plan International.” - Mother (FGD Agojo)	Mothers FGD (2)

Many fathers mentioned different ways that they support breastfeeding mothers including providing food and emotional support. Fathers said that they should make sure the breastfeeding mother has something to eat and that she feels happy. They also reported that they can assist with household chores that are typically considered the role of the wife such as helping clean the home and cultivating the garden as a way to support breastfeeding.

*“Your role is to provide the food for the mother who is breastfeeding, when you increase the diet for the wife the baby will breastfeed very well.” - Father (FGD Ayilo-I)*

*“Make her feel happy and loved.” - Father (FGD Ayilo-I)*

*“A husband is supposed to help his wife by taking part in cleanliness of home.” - Father (FGD Ayilo-I)*

Additionally, participants mentioned community support and support from other organizations as facilitators of breastfeeding. Community support included help from relatives, such as grandmothers, and also from neighbors. Medical Teams International (MTI) and Plan International, partner agencies of the United Nations High Commissioner for Refugees (UNHCR), were mentioned as the NGOs providing support to breastfeeding mothers in the area.

*“In our homes grandmothers do provide mothers with food for cooking so that she produce more breastmilk for the child.” – Mother (FGD Agojo)*

*“And also some neighbors do support mother with providing them firewood and drinking water.” - Mother (FGD Agojo)*

*“They were being supported by the partners MTI and Plan International who were giving out flour for porridge.” - Mother (FGD Agojo)*

### Barriers

Identified barriers were organized into four themes: knowledge, physical, socioeconomic, and psychosocial (Table 4). Both mothers and fathers reported that infants under six months could be fed supplemental feedings that include powdered milk diluted with water, cow’s milk, juice or formula. Also, some mothers discussed that breastmilk is not sufficient for sick infants and therefore, breastfeeding should be supplemented with other liquids.

**Table 4:** Barriers to breastfeeding

**Themes**	**Codes**	**Quotations **	**Data Source (# times mentioned)**
**Physical barriers**	Mother is sick	“Sometime the mother was sick and stop breastfeeding.” - Mother (FGD Agojo) “When the mother is sick and has been advised to stop the baby from breastfeeding.” - Mother (FGD Nyumanzi) “When the mother is sick, the baby will not be allowed to breastfeed.” - Mother (FGD Nyumanzi) “Another reason is when the mother has developed breast cancer a baby is not supposed to breastfeed.” - Father (FGD Ayilo-I) “Also when the mother is infected with HIV/AIDS then the baby is not supposed to be breastfed that is why the baby may not be given breastmilk.” - Father (FGD Ayilo-I) If the mother is sick then she cannot breastfeed the kid.” - Father (FGD Ayilo-I) “If the mother is sick she will not breastfeed.” - Father (FGD Ayilo-I)	Fathers FGD (5) Mothers FGD (4)
	Mother has died	“Sometimes the biological mother died.” - Mother (FGD Agojo) “The mother the baby might have died and therefore the alternative is supplementary feeding.” - Father (FGD Ayilo-I)	Fathers FGD (1) Mothers FGD (1)
	Perceived milk insufficiency	“Sometimes there is no breastmilk for the baby.” - Mother (FGD Agojo) “There will be no breastmilk.” - Mother (FGD Agojo) “When a baby is four months old and the mother does not produce enough milk they should be given other feedings.” - Mother (FGD Nyumanzi) “When the wife has produced twins extra feeding should be added on top of breastmilk because the milk from the mother is not enough for the two babies.” – Father (FGD Ayilo-I)	Fathers FGD (2) Mothers FGD (4)
	Breastfeeding difficulties	“They did not start giving breastfeeding on time that is why no breastmilk is coming.” - Mother (FGD Agojo) “When the baby doesn’t breastfeed well he or she should be given extra feedings.” - Father (FGD Ayilo-I) “When a baby is born and refuses to breastfeed that child is given the supplementary feeding.” - Father (FGD Ayilo-I)	Fathers FGD (2) Mothers FGD (2)
**Socioeconomic barriers**	Mother is working	“Other mothers are working class.” - Mother (FGD Agojo)	Mothers FGD (1)
	Educated mother uses other milk	“For educated mothers they can say they will not feed their child on breastmilk they will use other milk.” - Mother (FGD Agojo)	Mothers FGD (1)
**Knowledge barriers**	Breastfeeding should stop at 3 months	“Weaning a baby can sometime reach 3 months if the baby has no access to supplementary feedings and when it is naturally weak.” - Mother (FGD Nyumanzi)	Mothers FGD (1)
	Infant under 6 months should eat and drink items other than breastmilk	“When the mother produces less milk, then the parent buys the powder milk and dilute it with the water and give it to the baby they also buy Blueband [margarine] for the baby.” - Mother (FGD Nyumanzi) “The parent buys the powder milk and dilute it with the water.” - Mother (FGD Nyumanzi) “It can be after 3 months.” - Father (FGD Ayilo-I)	Fathers FGD (3) Mothers FGD (5)
	Sick baby needs more than breastmilk	“You try to feed a sick baby with milk.” - Mother (FGD Nyumanzi) “I can provide porridge for the sick baby.” - Mother (FGD Nyumanzi) “I can give a sick child some soft drinks the ones that he might want.” - Mother (FGD Nyumanzi)	Mothers FGD (4)
**Psychosocial barriers**	Fighting with husband	“When the wife is not happy at home for example she might have fought with the husband and she may refuse to breastfeed the child.” - Father (FGD Ayilo-I)	Fathers FGD (2)
	Fear of pain	“The other young woman who has just started giving birth may fear to breastfeed the baby she may feel painful to breastfeed.” - Father (FGD Ayilo-I)	Fathers FGD (1)
	Mother has mental health issues	“The mother is mentally challenged.” - Father (FGD Ayilo-I)	Fathers FGD (2)

*“When the baby is sick and has become very weak and although has not reached the required age, breastfeeding should be supplemented with other liquids such as milks and drinks.” - Mother (FGD Nyumanzi)*

*“It is to sustain the baby until 6 months and that is why the baby is given milk and blueband [margarine].” - Mother (FGD Nyumanzi)*

*“A baby is given cow’s milk before reaching 6 months.” - Nyumanzi (Mothers FGD)*

*“Other supplements are juices.” - Mother (FGD Nyumanzi)*

*“A baby is given infant formula such as Nan1 and Nan2 for their feeding.” - Father (FGD Ayilo-I)*

*“When you have cow’s milk, other parts of the milk is skimmed for butter and ghee and should be the supplement for the feeding of the baby.” – Father (FGD Ayilo-I)*

Many participants identified that when the mother is sick, there will be a disruption in breastfeeding. Mothers and fathers reported that sick mothers may have been advised to stop breastfeeding or believe that the disease may transmit through breastmilk.

*“When you are not healthy, you will not be allowed to breastfeed your baby.” - Mother (FGD Nyumanzi)*

*“When the mother is sick, the baby is supposed to stop breastfeeding so not to transmit the disease from the mother to the child.” - Father (FGD Ayilo-I)*

Mothers and fathers both reported that mothers may not produce enough breastmilk and experience other breastfeeding difficulties that lead them to stop breastfeeding. They stated that sometimes if a mother does not initiate breastfeeding early, she has difficulties breastfeeding or that a child may not breastfeed well.

*“Sometimes the breastmilk is not coming.” – Mother (FGD Agojo)*

*“It’s because the mother may lack enough breastmilk.” - Father (FGD Ayilo-I)*

*“The reason might be the mother have started to give breastmilk late after she has given birth.” – Mother (FGD Agojo)*

*“When a baby is born and refuses to breastfeed from the mother that child is given the supplementary feeding.” - Father (FGD Ayilo-I)*

Only a few mothers discussed barriers related to socioeconomic factors, such as working status and education. Working outside the home or higher levels of education were identified as being factors that hinder breastfeeding.

*“Other mothers are working class.” – Mother (FGD Agojo)*

*“For educated mothers they said they will not feed their child on breastmilk and they will use other milk.” - Mother (FGD Agojo)*

A few fathers mentioned psychosocial barriers such as marital conflict, mothers’ fear of pain during breastfeeding, and maternal mental health issues. Marital conflicts may lead to the mother temporarily leaving the home without her child, thus she stops breastfeeding.

*“Some women when they fight with the husband at home, they may decide to go to their mother’s home and leave the child with the husband.” - Father (FGD Ayilo-I)*

*“The young woman who has just started giving birth may fear to breastfeed the baby and she may feel pain while breastfeeding.” - Father (FGD Ayilo-I)*

*“When the mother is not normal like she is mad, then the baby should be fed with alternative feeding because she may even harm the baby so it is better to stop the baby from breastfeeding.” - Father (FGD Ayilo-I)*

## Discussion

### Facilitators

Both mothers and fathers mentioned beliefs and knowledge about breastfeeding benefits that were identified facilitators of breastfeeding. Participants discussed that breastmilk was nutritious, would make the baby grow to be smart, and would protect against disease as well as promote healthy growth. These findings are consistent with previous qualitative studies in Nigeria [16], Ghana [17], and Zimbabwe [18] which found that beliefs or knowledge about breastfeeding were facilitators of breastfeeding. Knowledge is an important part of behavior change and is an aspect of social and behavior change communication (SBCC) interventions [19]. The use of SBCC for improving breastfeeding practices has been widely shown to be effective across many countries [19]; however, evidence of the effectiveness of SBCC in protracted settlements and among refugees is limited. Because beliefs and knowledge were facilitators of breastfeeding in this study, it is important that interventions aiming to improve breastfeeding consider these two individual factors. Furthermore, training healthcare staff and community health workers to provide and communicate the benefits of breastfeeding to mothers during their antenatal and postnatal visits would be beneficial.

Fathers described different ways that they support breastfeeding mothers including contributing to household chores, procuring food, and providing emotional support.

Likewise, in Zimbabwe, the presence of a spouse who assisted with chores was a facilitator of breastfeeding [18] and in Ethiopia, fathers supported breastfeeding mothers by providing and contributing to meal preparation [20]. A recent systematic review revealed different ways fathers’ were involved in supporting breastfeeding mothers, including verbal encouragement and helping with household and child care responsibilities [21]. However, none of these studies were conducted among refugees in protracted settlements where fathers may not be present in the household for a variety of reasons including working in their home country or death during conflict. Additionally, a review of 28 projects in 20 low- and middle-income countries did not find consistent associations between male engagement and increases in breastfeeding and noted that there is currently a lack of evidence to make broad recommendations about targeting male engagement to improve breastfeeding [22]. Furthermore, reported defined gender roles among South Sudanese indicate that fathers are responsible for financially providing for their families while the mothers are responsible for household chores [23]. Therefore, future research may consider a more in-depth analysis of local gender norms, fathers’ presence in the protracted settlements, the level of influence they have in breastfeeding decisions, and the type of support they can offer. This information could inform whether father involvement would be an effective factor in supporting breastfeeding. A few participants mentioned community support as a facilitator of breastfeeding. Participants discussed how breastfeeding mothers receive help from relatives, such as grandmothers, and from neighbors. Interestingly, the type of support mentioned included material support such as food, firewood, and drinking water. Similar results have been found among refugee mothers in Syria; those who did not receive social support from relatives stopped breastfeeding [23]. Furthermore, in Ethiopia, breastfeeding mothers received support from grandmothers; however, the support differed in that grandmothers in Ethiopia also provided childcare and housework assistance for breastfeeding mothers [20]. A systematic review that assessed grandmothers’ roles in breastfeeding found that grandmothers can be influential in breastfeeding decisions and recommended that interventions aiming to improve breastfeeding should consider including grandmothers [24]. Future research might explore the family dynamics among refugees living in protracted settlements to better understand the role and influence of grandmothers on breastfeeding in a new environment.

Organizations such as Medical Teams International (MTI) and Plan International were cited as the NGOs providing support to breastfeeding mothers. Plan International provided breastfeeding education and both MTI and Plan International gave flour for porridge for breastfeeding mothers. While NGOs have generally not been mentioned as facilitators of breastfeeding in previous literature, in the context of protracted settlements, the presence of partners and NGOs with breastfeeding initiatives may be more common and might be an important avenue for improving breastfeeding practices among refugees. Interestingly, participants did not mention support from community health workers, medical staff, or peers as facilitators to breastfeeding. Support from peers has been shown to be an effective way to promote breastfeeding [25]. A systematic review and meta-analysis of the effectiveness of community-based peer support for mothers to improve breastfeeding practices, reported that in low- and middle-income countries, community-based peer support increased exclusive breastfeeding, compared to the standard care [26]. Interventions, facilitated by NGOs, or in partnership with the local health care system, involving the use of peers may provide efficient, cost-effective, and sustainable ways to improve breastfeeding among refugees living in protracted settlements.

### Barriers

While knowledge of breastfeeding benefits was an identified facilitator, the lack of knowledge of breastfeeding recommendations was a barrier. Many participants, both mothers and fathers, stated that infants under six months could receive supplemental feedings that included items such as powdered milk diluted with water, cow’s milk, juice, formula, or margarine. Introducing complementary feedings before six months and poor rates of exclusive breastfeeding have been reported previously in this area [10]. These results highlight the need for continued emphasis on education about infant and young child feeding practices at antenatal care, place of delivery, and postnatal care.

Breastfeeding difficulties and inadequate breastmilk supply were among the two main physical barriers preventing mothers from breastfeeding. Both mothers and fathers reported that mothers may not be producing enough breastmilk or may experience issues where the child is not breastfeeding well. While perceived low milk supply has been documented in many different contexts and is a common barrier to breastfeeding [20, 22, 27], it is uncertain if maternal perception of inadequate breastmilk is accurate. In order to overcome this barrier, it would be important for mothers to receive education about maternal factors that influence breastmilk supply and how to identify inadequate production. Ideally, such discussions would be best during antenatal care so that mothers are equipped to identify these issues early in their breastfeeding experience. Furthermore, hospitals and other places of delivery may consider training their staff on solutions to common lactation issues that mothers may face during the postnatal period, as well as expansion of community-based support for mothers who lack access to healthcare facilities.

Socioeconomic factors such as working status and education level were barriers mentioned by a few mothers. Despite only 14.3% of mothers working outside the home, this factor was identified as being a barrier to breastfeeding. A systematic review of factors influencing breastfeeding that included 25 studies from 19 countries found that in the majority of the studies, maternal employment outside the home was negatively associated with exclusive breastfeeding [28] which also is consistent with findings in more recent studies [20, 22, 27]. Overcoming this barrier may be very challenging, depending upon the type of the mother’s work outside the home. More information is needed from breastfeeding mothers about their specific barriers to work outside the home. Policies for working mothers may need to be implemented in both formal and informal settings, to allow them to bring their child to work and to allow time throughout the day for breastfeeding. Implementing policies for breastfeeding mothers in informal sectors may be more difficult and may require the influence of strong governmental policies.

The finding that mothers who have more education will chose other milk for their infants is consistent with a recent study in Uganda where mothers felt that if they have enough money, they should not allow their babies to breastfeed [29]. However, other studies have reported that higher education levels are often positively linked to breastfeeding [30–32], and low maternal education has been identified as a risk factor for suboptimal breastfeeding practices [33, 34]. Furthermore, a recent systematic review of 81 low- and middle-income countries found that compared to women with primary, secondary or higher education, women with no formal education had poorer adherence to breastfeeding indicators [35]. The inconsistent findings and variations across countries in the association between education level and breastfeeding may highlight specific cultural or societal beliefs influencing mothers. In a recent study in Uganda, participants reported a belief that breastfeeding was for poor women who could not afford an alternative [29].

In the context of refugees in settlements, formula is often one of the first donations to arrive in settlements and its’ donations remain unregulated. It has been noted that mothers who receive donations of formula may perceive it to be superior to breastmilk, and thus formula donations may serve as an impediment to exclusive breastfeeding among refugees in settlements. This reemphasizes the need for policies to regulate and control wide-spread donations of formula in refugee settlements, as well as provide information and support to breastfeeding mothers [36]. Combined with improvement in policies, future research is needed to better understand why mothers with presumably more knowledge about the benefits of breastfeeding may prefer breastmilk substitutes over breastfeeding. Further understanding of these beliefs would inform interventions designed to improve breastfeeding among mothers regardless of their educational level.

Only a few fathers discussed psychosocial barriers such as marital conflict, mothers’ fear of pain during breastfeeding, and maternal mental health issues. Marital conflict, in the form of intimate partner violence (IPV) [37–39] and maternal mental health issues [40–42] have a negative influence on breastfeeding. Refugees are at increased risk for mental health issues [43] and refugees living in Uganda’s protracted settlements often suffer from psychosocial difficulties [44–49].

Interestingly, none of these psychosocial barriers were reported by the mothers in the FGDs. Possibly the mothers felt uncomfortable disclosing marital conflict or mental health issues as stigma regarding mental health problems has been documented among refugees [50] and specifically, among the South Sudanese population [51]. These results highlight the importance of including FGDs for fathers as well as mothers because both parents may provide additional insights essential to improving breastfeeding practices. Furthermore, screening for IPV and maternal mental health during antenatal and postnatal care may be imperative to improve breastfeeding among mothers living in these protracted settlements. However, for maternal mental health issues to be addressed adequately, reducing the stigma around these types of issues will be necessary so that individuals are willing to access available resources.

A psychosocial factor not mentioned by fathers or mothers is self-efficacy or the confidence in ability to breastfeed which is reportedly a known facilitator of breastfeeding [32–56]. A systematic review and meta-analysis that included 24 randomized controlled trials from 14 countries found that theory-based educational interventions have been effective in improving breastfeeding self-efficacy and breastfeeding rates at 6 months [57]. Assessing self-efficacy concerns of refugee women could inform the design of effective breastfeeding interventions among this population.

### Limitations

Although participants from different protracted settlements were included, the results may not be generalizable across all refugees from other districts. Furthermore, parents who were willing to participate may not be representative of the population. Lastly, limitations arising from social desirability bias were possible due to the nature of qualitative research and may explain some of the differences between the responses of mothers and fathers.

## Conclusion

This study revealed the complexity of the barriers to breastfeeding faced by mothers living in protracted situations. Barriers included individual knowledge of breastfeeding recommendations, physical challenges, socioeconomic status, and education level, as well as psychosocial barriers. To tackle these wide-ranging barriers, interventions and policies guided by the socioecological model may best address the concerns discussed by participants. Furthermore, because facilitators of breastfeeding included beliefs and knowledge about benefits, antenatal staff should continue to deliver messaging about the benefits of breastfeeding. Support from fathers, the community, and NGOs were facilitators; therefore, these support networks are likely key aspects of an effective strategy to improve breastfeeding among mothers in protracted settlements.

## Data Availability

The data can be made available upon request.

## Abbreviations

FGD: Focus group discussion
IPV: Intimate partner violence
LWF: Lutheran World Federation
MTI: Medical Teams International
NGO: Non-governmental organization
SBCC: Social and behavior change communication
UNHCR: United Nations High Commissioner for Refugees
WHO: World Health Organization
